# From Consultation to Collaboration: A Ladder of Participation for Older Adults in Research

**DOI:** 10.1093/geroni/igaf122.430

**Published:** 2025-12-31

**Authors:** Caitlin Coyle

**Affiliations:** University of Massachusetts Boston, Boston, Massachusetts, United States

## Abstract

Assumptions about what older adults want and need can drive research and planning such that systemic ageism is propagated within systems. Representation of older adults in investigations and initiatives is needed so that policies, programs and interventions developed out of research are in alignment with the needs and preferences of older people. The aim of this article is to describe the creation of a ladder of participatory opportunities for older people to contribute to a needs assessment process. This process allows for co-researchers to objectively shape of environments and services for their peers. Data comes from 5 community needs assessment projects and the accumulation of 4 older adult research associates. Data were collected via surveys, key informant interviews, and focus groups. We describe how participatory principles were enacted, the involvement of older adults in research design, data collection, and dissemination. As well, we describe the process by which older people can move from a project-based participation to becoming a member of an academic research team. Finally, we discuss the ways in which a community inclusive multi-method strategy can optimize resource allocation, identify pathways to more effective policymaking that is matched to the needs and interests of its oldest residents, and lead to unanticipated benefits. Consistent and equivalent collaboration between researchers and older people can provide critical context and perspectives of the those challenged by their aging circumstances and distribute power to older people for the purposes of being active builders of their environments.

